# Evaluation of vaping cessation infographics among e-cigarette users: A cross-sectional, mixed-methods study

**DOI:** 10.18332/tpc/214725

**Published:** 2026-01-23

**Authors:** Hongying Daisy Dai, Chenxi Shi, Sara Reyes, Summer Woolsey

**Affiliations:** 1 College of Public Health, University of Nebraska Medical Center, Omaha, United States; 2 College of Osteopathic Medicine, Kansas City University, Joplin, United States

**Keywords:** current e-cigarette users, vaping cessation, infographics, vaping cessation motivation

## Abstract

**INTRODUCTION:**

This rise in vaping has become a significant public health concern. This mixed-methods study aims to assess the impact of vaping cessation infographics on different cessation motivation and gather feedback regarding how the infographic could be further improved.

**METHODS:**

Current e-cigarette users completed an online cross-sectional survey in January 2022 through Prolific^®^. Participants evaluated a vaping cessation infographic. Eligible participants were adults aged 19–64 years, US residents, fluent in English, and current e-cigarette users. Participants completed a questionnaire with questions regarding demographics, e-cigarette use, and assessment of the liking of infographics. Poisson regressions were conducted to assess the associations between infographic liking and perceived vaping cessation importance, readiness, and commitment. The open-ended feedback provided in the online survey on the infographic was assessed using content analysis.

**RESULTS:**

Among 361 participants who were presented with the cessation infographic, 85.9% of them rated the infographic favorably. The liking of the cessation infographic (e.g. excellent vs fair/poor) was associated with greater perceived quit importance (incidence rate ratio, IRR=2.0, p<0001, quit commitment (IRR=1.6, p=0.005), and readiness to quit (IRR=1.9, p=0.001). The analysis of open-ended feedback resulted in several themes. Participants appreciated the detailed coping strategies, ease of understanding and accessibility, timelines and expectations, support and encouragement, comprehensive information, youth-focused content, and visual and structural appeals. Areas identified for improvement included health information and risks, design and presentation, target audience and content, support and resources, and user engagement.

**CONCLUSIONS:**

This study highlights the need for tailored vaping cessation interventions. The infographic presented in this study resonated more with African American and Hispanic participants, who reported higher favorability than White participants.

## INTRODUCTION

E-cigarettes are the second most consumed tobacco product in the United States, behind cigarettes^1,2^. In 2021, 46 million US adults reported consumption of tobacco products in the National Health Interview Survey; at least a quarter of them reported e-cigarette use alone or concurrently with other tobacco products^1^. E-cigarette use is particularly higher among current and former smokers (vs never smokers), young (vs old) adults, and those with monthly depressive episodes (vs without)^3,4^. Adults use e-cigarettes for a variety of reasons. Among older adults, current and former smokers make up the majority of e-cigarette users, with smoking cessation as their primary intention^5,6^, while increased popularity among younger e-cigarette users is drawn by flavoring and curiosity and they are more likely to be non-smokers^3,6,7^. Although e-cigarettes are generally considered less toxic than regular cigarette smoking^8^, the role of e-cigarette use in smoking cessation is inconclusive^9^, and the U.S. Food and Drug Administration (FDA) has not approved any e-cigarette products to help people quit smoking^8^. Additionally, e-cigarette aerosol contains a number of potentially toxic substances (e.g. carbonyl compounds, heavy metals, and flavorings such as diacetyl)^10^ and e-cigarettes are associated with an increased risk of respiratory symptoms^11,12^. The ultimate goal for dual users of e-cigarettes and combustible cigarettes or exclusive e-cigarette users is to quit all tobacco use, in order to minimize potential adverse health risks^13^.

Both tailored and non-tailored patient education materials have been shown to help quit smoking^14,15^. Although similarities exist between vaping and smoking cessation, the difference in user demographics and reason for use all require new vaping-specific cessation programs targeting these unique groups^16^. Previous research highlighted addictive flavors, ease of use, and poor self-awareness as unique barriers to quitting vaping^16^, thus interventions that address these barriers will be likely to improve vaping cessation outcomes. Historically, text messages, web-based resources, or voice calls have been utilized to deliver cessation materials^17-19^. However, these routes were only variably effective in targeting the large population, including young adults. In the past, the inclusion of graphics in smoke cessation messages was shown to be more effective than texts alone^20^.

With print or digital handouts that include fact-based statements and images of health risk information, a brief health education can be an effective strategy for enhancing health perceptions and reaching a broad audience^21,22^. The Theory of Planned Behavior postulates that the likelihood of an individual engaging in a healthy behavior (e.g. vaping cessation) is associated with attitude, subjective norm, and perceived behavioral control^23^. Brief education on vaping cessation can help shift perceptions and attitudes toward e-cigarette use, enhancing knowledge and increasing the intention to pursue cessation in the future. A prior study conducted on current tobacco product users showed that brief intervention through education handouts increased participants’ knowledge and modestly reduced their intentions for future use^24^. However, to the best of our knowledge, no studies have examined the e-cigarette users’ perceptions of an infographic on vaping cessation and whether the liking of the infographic is associated with vaping cessation motivation.

This study examined the feedback of adult e-cigarette users on a vaping cessation infographic through a mixed-methods study. We conducted a quantitative analysis to assess: 1) the liking of vaping cessation infographics and whether the liking differed by key demographic and vaping behavior factors; and 2) whether the liking was associated with vaping cessation motivation among current e-cigarette users. We also qualitatively reviewed open-text comments provided by e-cigarette users regarding what they liked most about the vaping cessation infographic and how the vaping cessation infographic could be improved.

## METHODS

### Study design

We conducted a cross-sectional online survey of targeted convenience sampling of e-cigarette users in the United States in January 2022 through Prolific^®^, using self-administered questionnaires to collect data. Prolific^®^ is an online crowd-sourcing recruitment platform that includes over 47000 diverse US panel members from all 50 states for psychological and behavioral research^25^. Prolific can provide a large national sample with pre-screened filters at a relatively low cost. The platform complies with General Data Protection Regulation (GDPR) standards^25^, and data from Prolific have been used in other substance use research studies, demonstrating high data quality^26-28^. The survey design methodology was described in a previous study^28^. Briefly, survey participants were selected if they met the following criteria: 1) adults aged 19–64 years, 2) current US state of residence, 3) fluent English speakers, 4) current e-cigarette users (i.e. those who reported using e-cigarettes some days or every day); and 5) Prolific approval rate >90% among previous studies for quality control purpose. Written consent was obtained from participants after being informed of the data confidentiality and the voluntary nature of the survey. All participants were compensated for completing the survey based on the recommended price from Prolific (about $5.10 for a 30-minute survey). The average survey completion time among all participants was 13.8 minutes. Quality control measures included a manually conducted review to identify fraudulent or duplicate responses, embedding a data quality check question within the survey, incorporating a randomly generated completion code, and mandatory verification steps to prevent automated completions. This study was approved by the University of Nebraska Medical Center (UNMC) Institutional Review Board.

### Infographics

The UNMC research team developed a vaping cessation infographic using a multi-faceted approach. This process included a comprehensive literature review to identify evidence-based cessation methods, consultation with cessation experts for practical tips, and a curated summary of useful resources. Additionally, the team utilized visual design principles to enhance engagement and readability, and conducted iterative revisions based on pilot testing to optimize the infographic’s effectiveness in supporting vaping cessation efforts. Our team has also established a long-term collaboration with the local tobacco coalition, Tobacco Education & Advocacy of Midland, to gather valuable inputs and effectively disseminate research findings to the community. The infographic focuses on the harmful effects of e-cigarette use, vaping cessation motivation, triggers, and coping strategies, along with resources to support the cessation (Supplementary file Figure 1). A total of 361 current e-cigarette users were presented with the infographics, and several questions were asked about their views on the infographics and their expectations regarding vaping cessation. They were also given the opportunity to provide comments in a free-response format.

### Survey measures

The liking of the infographic was assessed by asking participants to evaluate the vaping cessation infographic on a 5-point Likert scale: 5=excellent, 4=very good, 3=good, 2=fair and 1=poor. Due to the small number of ‘poor’ and ‘fair’ responses, we recoded the variable on a 4-point scale by combing poor and fair so that: 1=poor/fair, 2=good, 3=very good, and 4=excellent. Four additional learning outcomes from the vaping cessation infographic were also asked, including: ‘I learned things I did not already know about e-cigarettes’, ‘I feel I would be more confident to say “No” if someone were to offer me an e-cigarette or invite me to vape’, ‘I will be less likely to vape now’ and ‘I can better understand hidden messages in vaping ads, especially those targeted at young people’, with 4-level response options ranging from ‘strongly disagree’ to ‘strongly agree’.

Vaping cessation motivation was adapted from previous smoking cessation studies^29,30^, and participants were asked about three separate questions regarding the importance of quitting (‘As of now, how important is it for you to quit or stop using e-cigarettes?’), the commitment to quitting (‘As of now, how committed are you to quit or stop using e-cigarettes?’), and the readiness to quit (‘As of now, how ready are you to quit or stop using e-cigarettes?’). Each question was presented with response options ranging from 0 (not at all important/committed/ready) to 5 (very important/committed/ready).

Write-in responses were provided by participants in regard to two separate questions: ‘What did you like most about the vaping cessation infographic?’ and ‘How could the vaping cessation infographic be improved?’.

### Covariates

*Sociodemographics*

Age in years (19–64 years), sex (males, females), race/ethnicity (Non-Hispanic [NH] White, NH Black, Hispanic, or NH Other [i.e. American Indian or Alaska Native, Asian, Native Hawaiian or Pacific Islander, Middle East or North African, or more than two races]), education (lower than high school/high school graduates, some college/college graduates), annual household income ($) (<25000, 25000–49999, 50000–74999, 75000–99999, ≥100000), personal financial situation (live comfortably, just meet or do not meet basic expenses), and sexual orientation (heterosexual or sexual minority).

*Substance use behaviors*

Current other tobacco use (i.e. cigarettes, big cigars, little cigars or cigarillos, hookah or water pipe, chewing tobacco, snuff, snus), and other substance use (i.e. including marijuana or hashish, one full drink of alcohol, any non-prescription drugs, and other illicit drugs); and 3) e-cigarette use behaviors such as vaping frequency (some days or every day), e-cigarette devices used most often in the past 30 days (i.e. vape pen or pen-like, JUUL or similar rechargeable devices with cartridge, disposable devices, or other), flavors used most often in the past 30 days (i.e. flavorless or tobacco flavored, mint or menthol, sweet, ice, and other), and past 6-month quit attempts (no vs yes). Potential confounders such as vaping frequency, past 6-month quit attempts, current other tobacco use, and other drug use were controlled in the analysis.

### Statistical analysis

Among the participants, 56.6% (n=133) identified as female, while 43.4% (n=102) identified as male. The majority of participants were Non-Hispanic White (67.4%; n=172). The overall mean score for liking the infographic was 2.7 (SD=1.0) on the range 1–4. Among females, the mean was 2.7 (SD=0.9), while among males it was 2.6 (SD=1.0). Across racial groups, Non-Hispanic Black participants reported the highest mean rating of infographic liking at 3.0 (SD=1.0), followed by Hispanic participants at 2.8 (SD=1.1). White participants reported a mean of 2.6 (SD=0.9), and those identifying as ‘Other’ reported a mean of 2.5 (SD=1.1).

After the descriptive statistics of the sample characteristics, the liking of vaping cessation infographic was presented, overall and by key factors (e.g. sex, race/ethnicity, and past 6-month quit attempt). Chi-squared tests were performed to detect any significant group differences. Next, multivariable regression models were conducted to examine associations between the liking of vaping cessation infographics, the independent variable and dependent variables measuring vaping cessation motivation (i.e. importance, commitment, and readiness) with the Poisson link function in modeling the count variables. Model selection along with Poisson regression and dispersion tests were performed using goodness-of-fit test. Incidence rate ratios (IRRs) with 95% confidence intervals (CIs), adjusting for sociodemographic covariates (i.e. age, sex, race/ethnicity, education level, financial situation, sexual orientation), e-cigarette use status (i.e. daily/some days, past quit attempts), and other tobacco and drug use. A p<0.05 (2-tailed) was considered as statistical significance. Statistics were calculated using SAS 9.4 (Cary, NC).

For the qualitative study, content analysis was used to evaluate feedback from the infographics. Two coders (SR and CS) independently reviewed the feedback regarding their views of vaping cessation infographics to identify themes. Team members then refined themes through group discussion. Cohen’s kappa coefficient was used to measure inter-coder reliability, and the final coded transcripts were reviewed by another author (HD) to ensure coding consistency and accuracy.

## RESULTS

The analytical sample included 361 current e-cigarette users who were presented with the vaping cessation infographic and asked to complete additional questions. Sample characteristics are presented in Supplementary file Table 1. The average age of the participants was 34 years (SD=11.4), and 58.3% were females. The sample is made up of a population diverse and nationally representative in education (high school or lower 15.5%, some college 45.2%, college graduate 39.3%), racial representative (NH White 72.3%, NH Black 5.6%, Hispanic 10.6%, Other 11.5%), sexual orientation (heterosexual 75.1%, sexual minority 24.9%), and income ($) (<25000: 17.5%, 25000–49999: 26.7%, 50000–74999: 21.9%, 75000–99999: 18.9%, ≥100000: 18.6%). Additionally, less than half (42.7%) reported current usage of other tobacco products, the majority of current e-cigarette users reported some days use (73.1%) and concurrent other drug use (79.8%), and 30% reported past 6-month vaping quit attempts.

Assessment of the liking of the vaping cessation infographic is presented in Table 1. Overall, 85.9% of participants rated infographic favorably (14.1% as excellent, 36.7% as very good, 26.7% as good). The mean of the liking rating was overall very favorable [mean=2.7 (SD=1.0), range 1–4). The liking rating was not significantly different between the sexes [females: 2.7 (0.9), males: 2.6 (1.0), p=0.58] and past 6-month quit status [‘No’: 2.6 (SD=1.0), ‘Yes’: 2.9 (1.0), p=0.10]. There were also no differences among racial minorities (p=0.10) such as Hispanics [2.9 (SD=1.0)], Non-Hispanic Black [2.8 (SD=1.0)], groups that classify as White [2.6 (SD=1.0)] or Other [2.6 (SD=1.0)].

**Table 1:** Liking of infographics, overall and by key factors, United States, January 2022 (N=361)

*Liking of infographics (score)*	*Overall n (%)*	*Sex*			*Race/ethnicity*					*Past 6-month quit attempts*		
		*Female n (%)*	*Male n (%)*	p*	*NH-White n (%)*	*NH-Black n (%)*	*Hispanic n (%)*	*Other n (%)*	p*	*No n (%)*	*Yes n (%)*	p*
Fair/poor (1)	50 (14.1)	24 (11.9)	25 (17.1)	0.58	38 (15)	3 (15.8)	3 (8.1)	6 (15)	0.10	39 (15.7)	11 (10.5)	0.10
Good (2)	95 (26.8)	56 (27.7)	39 (26.7)		65 (25.6)	2 (10.5)	12 (32.4)	15 (37.5)		73 (29.4)	21 (20)	
Very good (3)	130 (36.7)	76 (37.6)	52 (35.6)		100 (39.4)	9 (47.4)	8 (21.6)	10 (25)		91 (36.7)	39 (37.1)	
Excellent (4)	79 (22.3)	46 (22.8)	30 (20.5)		51 (20.1)	5 (26.3)	14 (37.8)	9 (22.5)		45 (18.1)	34 (32.4)	
Mean score (SD)	2.7 (1)	2.7 (0.9)	2.6 (1)		2.6 (1)	2.8 (1)	2.9 (1)	2.6 (1)		2.6 (1)	2.9 (1)	

* Chi-squared tests were performed to detect any significant group differences. NH: Non-Hispanic.

Results of additional learning outcomes (e.g. learning e-cigarettes, saying no to e-cigarettes, being less likely to vape now, and having a better understanding of hidden information) are given in the Supplementary file.

In Table 2, associations of infographics liking with various vaping cessation motivation are presented. Overall, e-cigarette users scored higher for perceived quit importance, commitment, and readiness when their liking of the infographics was higher. For instance, the mean (SD) of the perceived quit importance was 1.2 (1.6), 1.9 (1.3), 2.3 (1.5), and 2.7 (1.8) when participants rated the infographic liking as fair/poor, good, very good, and excellent, respectively. In the multivariable regression, participants scaled significantly higher when asked about quit importance among those who gave ratings such as very good (IRR=1.9; 95% CI: 1.4–2.5, p<0.0001) and excellent (IRR=2.0; 95% CI: 1.5–2.7, p<0.0001) on the infographics compared to those who rated the infographics as fair/poor. Similarly, higher quit commitment is also associated with very good (IRR=1.9; 95% CI: 1.4–2.6, p=0.0001) and excellent (IRR=1.6; 95% CI: 1.2–2.3, p=0.0049) ratings of the infographics. Lastly, the groups demonstrate higher quit readiness (very good: IRR=2.1; 95% CI: 1.5–3, p<0.0001, excellent: IRR=1.9; 95% CI: 1.3–2.7, p=0.0012) when rating the infographic favorably compared to those rating it as fair/poor.

**Table 2:** Associations of infographics liking and vaping cessation motivation, United States, January 2022 (N=353)

*Liking of infographics*	*Quit importance*			*Quit commitment*			*Quit readiness*		
	*Scale ^a^*	*IRR ^b^*	*p ^b^*	*Scale ^a^*	*IRR ^b^*	*p ^b^*	*Scale ^a^*	*IRR ^b^*	*p ^b^*
Fair/poor (ref.)	1.2 (1.6)	1		1.0 (1.5)	1		0.8 (1.2)	1	
Good	1.9 (1.3)	1.6 (1.1–2.1)	0.0047	1.3 (1.1)	1.3 (0.9–1.9)	0.1173	1.2 (1.1)	1.5 (1–2.2)	0.0273
Very good	2.3 (1.5)	1.9 (1.4–2.5)	<0.0001	1.8 (1.4)	1.9 (1.4–2.6)	0.0001	1.7 (1.5)	2.1 (1.5–3)	<0.0001
Excellent	2.7 (1.8)	2 (1.5–2.7)	<0.0001	1.8 (1.7)	1.6 (1.2–2.3)	0.0049	1.7 (1.8)	1.9 (1.3–2.7)	0.0012

a Ranging from 0 (not at all important/committed/ready) to 5 (very important/committed/ready).

b Multivariable regression models were conducted to examine associations between the liking of vaping cessation infographics and vaping cessation motivation (i.e. importance, commitment, and readiness) with the Poisson link function in modeling the count variables. IRR: incidence rate ratio.

Additional evaluation of cessation infographics is presented in Supplementary file Table 2. After reviewing the infographics, participants rated the highest ‘learning e-cigarettes’ [mean score (SD) = 2.6 (0.8), range 1–4], followed by ‘better understand hidden information’ [2.6 (SD=0.8)], ‘say no to e-cigarettes’ [2.3 (SD=(0.8)], and ‘less likely to vape now’ [2.0 (SD=0.8)]. Daily e-cigarette users were more likely than some days users to report ‘agree’ or ‘strongly agree’ on ‘say no to e-cigarettes’ and ‘less likely to vape now’ after reviewing the infographics.

Table 3 summarizes key themes that were identified in the responses when participants were asked about what they liked. Seven themes emerged among 240 comments, including detailed coping strategies, ease of understanding and accessibility, timelines and expectations, comprehensive information, youth-focused content, and visual and structural appeal. For instance, participants appreciated the sections on the infographics that give guidance to vaping users on what to do during quitting when they have cravings (e.g. *‘The coping mechanisms to help reduce cravings’* under [Detailed coping strategies]) and what to expect during that period (e.g. *‘The timeline that shows what to expect when stopping nicotine consumption. I’ve felt a lot of the symptoms’* under ‘Timelines and expectations’). Participants also mentioned that the information was easy to understand (e.g. *‘The infographic offers easy-to-understand information and potential strategies for reducing dependence on nicotine’* under [Ease of understanding and accessibility]), was comprehensive as the infographics covered a wide variety of topics from nicotine side effects to addiction self-assessment (e.g. *‘I liked the sheer amount of information inside of the two pages. I feel like most people would learn more reading this PDF compared to trying to piece together information from various other sources’* under [Comprehensive information]). The infographics received good feedback for their visual appeal and easy-to-follow format as well (e.g. *‘It was well organized and gave straight forward information. It gave options for alternatives*’ under [Visual and structural appeal]).

**Table 3:** Participant feedback of what they liked about vaping cessation infographics, United States, January 2022 (N=361)

*Themes*	*Selected quotations*
**Detailed coping strategies**	*‘I like how it listed what to expect and steps on how to quit.’* *‘It offered alternatives to do when you want to vape.’* *‘The coping mechanisms to help reduce cravings.’*
**Ease of understanding and accessibility**	*‘The infographic offers easy-to-understand information and potential strategies for reducing dependence on nicotine.’* *‘The information is clear and very well distributed in this infographic. It looks great, and informs me of the dangerous effects* *of e-cigarettes.’* *‘The language used is not too complicated to understand. It’s simple yet informative.’*
**Timelines and expectations**	*‘The part about what to expect when you stop using nicotine.’* *‘The timeline that shows what to expect when stopping nicotine consumption. I’ve felt a lot of the symptoms.’* *‘The warnings about how you will feel when you go into nicotine withdrawal.’*
**Support and encouragement**	*‘This is concrete information that makes me feel hopeful about quitting and builds my arsenal for quitting. It’s empowering and truthful.’* *‘It gives me more of a strength and sense to quit … its different than anything ive ever seen.’* *‘I liked how non-judgmental it was.’*
**Comprehensive information**	*‘I like the information that was given on the broad scope of the effects are what the kind what contains nicotine and the effects of it’* *‘I liked the sheer amount of information inside of the two pages. I feel like most people would learn more reading this PDF compared to trying to piece together information from various other sources.’* *‘I mean, it’s hard to say that this isn’t a very detailed infographic covering most, if not everything I can think of regarding vaping and the risks or associations that follow.’*
**Youth-focused content**	*‘I like the way they separated the teens with their own specific “quit” support. In doing this, it gives them a better chance. Also because I started smoking in my teens. It was “cool” in the 70’s ... Had I just known then.’* *‘The infographic had lots of information especially for teen or users that are not as familiar with the vaping process and effects it can have. I really like that fact that there are several sources to help you quit, even an app which I plan to check out.’*
**Visual and structural appeal**	*‘It was well organized and gave straight forward information. It gave options for alternatives.’* *‘I really like how they use their wording to get your attention. The big “You got this!” had really caught my attention and made me want to look. I also like how they start right out of the gate and say vaping is dangerous.’*

Table 4 lists six themes that were identified by the participants when they were asked how the infographics could be improved (n=236 comments), including health information and risks, design and presentation, target audience and content, support and resources, content improvements, and user engagement. Many mentioned they want more information on the health impact of vaping (e.g. *‘I would go into detail about the long-term health risks benefits more’*), and how vaping compares to other tobacco products (e.g. *‘Discuss why it is better than smoking cigarettes’* under [Health information and risks]). Some also mentioned the addition of testimonies from past vape users who successfully quit to increase engagement (e.g. *‘The vaping cessation could add the story of someone who experience nicotine addiction and have them give their advice on how to avoid it.’* under [User engagement]). Additionally, some users have asked for additional resources such as a hot line or an online community where users can interact or resources where there are professionals to talk to (e.g. *‘Maybe offering a support hotline where you can call a number to talk to real people just to distract you when you feel the need to smoke’ or ‘Adding a link to relevant Reddit subreddits for support’* under [Support and resources]). While the majority of users found the infographics to be organized and boast their content, a very small subset of users mentioned how the layout and design could be improved (e.g. *‘Font, it’s too light makes it hard to read almost blurry like, not sure if my screen. Info on it is good’ or ‘Fewer words and more images/diagrams’* under [Design and presentation]).

**Table 4:** Participant feedback of what improvements they recommended about vaping cessation infographics, United States, January 2022 (N=361)

*Themes*	*Selected quotations*
**Health information and risks**	*‘I would go into detail about the long-term health risks benefits more.’* *‘Discuss why it is better than smoking cigarettes’* *‘Go into detail about how E-Vali works.’*
**Design and presentation**	*‘Font, it’s too light makes it hard to read almost blurry like, not sure if my screen. Info on it is good.’* *‘Fewer words and more images/diagrams.’*
**Target audience and content**	*‘A lot of material on quitting doesn’t resonate with me because it focuses on the idea of vaping to fit in or look cool, rather than using it as a coping mechanism.’* *‘I think it needs to not only appeal to young people but people of all ages and walks of life.’*
**Support and resources**	*‘Maybe offering a support hotline where you can call a number to talk to real people just to distract you when you feel the need to smoke.’* *‘Adding a link to relevant Reddit subreddits for support.’* *‘Links or referrals to outside sources.’*
**Content improvements**	*‘More details on what occurs during cessation.’* *‘More of why you should quit or not start.’* *‘more concise info on why its harmful, use solid facts, numbers, and statistics, use scarier imagery.’*
**User engagement**	*‘The vaping cessation could add the story of someone who experience nicotine addiction and have them give their advice on how to avoid it.’* *‘Add a FAQ section about vaping.’*

## DISCUSSION

This study provided insights into the effect of brief intervention via infographics on vaping cessation motivation. The infographic was delivered during an online survey to current e-cigarette users and provided information such as cessation timeline, support resources, coping strategies, scientific facts, etc. Our study found that the vast majority of participants rated the infographics favorably. This is important as it can contribute to a successful future cessation in several ways. In the study, the level of liking was associated with increased vaping cessation motivation, which included quit importance, quit commitment, and quit readiness. This observation was not unexpected. The infographics contain comprehensive information on vaping cessation, including what to expect and coping mechanisms. As reported previously in other literature, these motivation metrics are highly indicative of cessation success.

Vaping is an urgent public health concern as the number of users continues to rise, and the associated long-term health consequences are still to be established^8^. Currently, there are limited randomized-control studies of vaping cessation interventions. Recent studies using interactive text messages (‘This is Quitting’) show success in young adult and adolescent e-cigarette users^17,18^.

Studies have shown that individuals with positive cessation motivation tend to have a better outcome in cessation^30,31^.This includes cessation readiness, quit commitment, quit importance, etc. The importance of change and commitment to change are often viewed as key intermediate steps toward reducing cigarette smoking^29^. Previous studies show that smokers who are more ready to quit are more susceptible to interventions and subsequent quitting attempts^29,30^. Similarly, higher intention to quit is associated with continued abstinence at 6 months^29-32^. Seeing all the information in the infographics could have helped evoke a sense of self-efficacy and confidence, making them feel more equipped to pursue their cessation goals and believe that success is more likely. Moreover, many current e-cigarette users planned to quit vaping and had past quit attempts, including nearly one-third of participants in this survey, so it was likely that they found the information to resonate and provided solutions to the hurdles they might have encountered during their quitting journey and motivating them to re-attempt^33,34^. Liking also contributes to a successful cessation by encouraging engagement with the intervention and adhering to the program recommendations. All these factors combined are the required recipe for cessation success^17,30^.

The rise in vaping has occurred among various demographics, particularly among young people^2,4^. What makes developing an effective cessation challenging is the different intentions for use among each group^4,16^. Consistent with previous studies^19,35^, this study continues to demonstrate that tobacco cessation interventions should not be a ‘one-size-fits-all’ approach and require a tailored approach based on groups^5,6^. In this study, the targeted population was adult e-cigarette users, and the information presented in the infographics contained information specifically catering to this population. In response, the infographic contains information on how nicotine in e-cigarettes could be harmful and ways to curb nicotine withdrawal. Conversely, when addressing a different population of e-cigarette users, such as adolescents, it will include information addressing the vulnerability of marketing influence^28^ and peer pressures. Although there were no differences among racial minorities, it is important to continue looking at disparities. Engaging in racial monitoring in tobacco cessation has been challenging due to cultural relevance, limited access to resources, and targeted marketing^35,36^. Future randomized controlled studies can evaluate the effectiveness of the targeted infographics in vaping cessation among racial minority groups.

While most participants found the infographics to be comprehensive and sufficient, all the users were asked to provide areas for future improvement, and their feedback was qualitatively analyzed. Some users appreciated the simple and easy-to-read format, while others were advocating for more thorough information. A key suggestion was the creation of a forum where individuals attempting to quit vaping could ask questions and share advice. This forum could also serve as a platform for former vapers to discuss their own unique experience and provide tips that helped them succeed. In future developments, QR code integration could be considered to bring additional resources for users who are seeking deeper engagement or even a more interactive version of the infographics.

### Strengths and limitations

The strength of this study is the integration of quantitative and qualitative results in the evaluation of a brief infographic vaping cessation program by a diverse background of current e-cigarette users recruited from Prolific^®^, a nationwide online platform. However, this study has several limitations. First, this survey collected self-reported data that were only for a one-time participation, and no follow-up was performed to assess any changes in e-cigarette use. Self-reported data come with limitations including the possibility of information bias and misreporting e-cigarette use. Future studies should evaluate whether the infographic can lead to changes in vaping cessation intention and cessation outcomes. Second, this was a study that utilized targeted convenience sampling, and the study participants might not be a representative sample of all e-cigarette users in the United States. Finally, the cross-sectional study design precludes causal inference. It is possible that current e-cigarette users with low vaping cessation motivation (e.g. low readiness to quit) were more likely to rate the liking of infographics as unfavorable.

## CONCLUSIONS

A brief infographic delivered through the form of infographics was associated with vaping cessation motivation in current adult e-cigarette users. Additionally, user feedback was analyzed to determine new improvements that can be made to the infographics. Vaping cessation motivation and vaping cessation outcomes should be re-evaluated in follow-up studies to determine if the effect of the infographics persists. Additionally, with the increased need for vaping cessation among adolescents^37^, the impact of infographics on teen users could be explored.

## Supplementary Material



## Data Availability

The data supporting this research are available from the authors upon reasonable request.
