# Supplementary material for: Evaluation of vaping cessation infographics among e-cigarette users: A cross-sectional, mixed-methods study
Source: Tob Prev Cessat. 2026 Jan 23;12:10.18332/tpc/214725. doi: 10.18332/tpc/214725 (PMC12828820; doi:10.18332/tpc/214725)
Supplement: Supplementary file 1 [file TPC-12-03-s1.pdf]

Appendix Figure 1. Infographics Designed for Vaping Cessation (2-page)

Thinking about quitting vaping?

You Got This

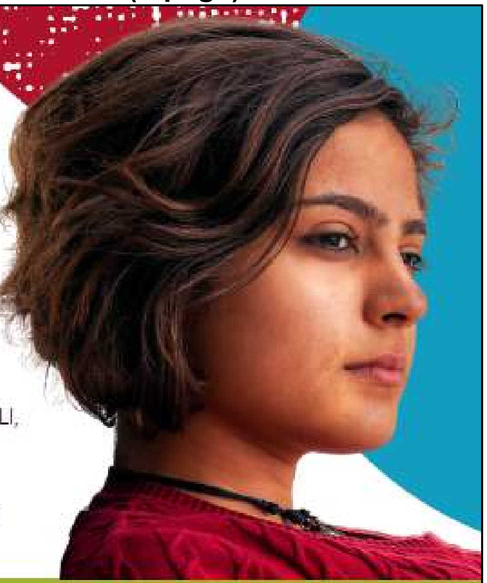

It's no secret, e-cigarettes are dangerous.

E-cigarettes may be advertised as safe alternatives to traditional cigarettes, but the truth is e-cigarettes are dangerous and can lead to adverse health effects such as lung damage, respiratory illness, nicotine addiction, and E-VALI, which can lead to death. Quitting vaping reduces your risk of cancer, heart attack, and can improve your lung function, blood circulation, and even improve your sense of smell and taste. With so much on the line, now is the best time to quit vaping.

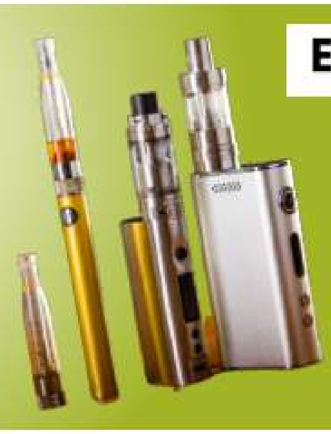

E-Cigarettes can lead to Nicotine Addiction

99% of all e-cigarettes contain nicotine. Once inhaled, nicotine travels to the brain in as quickly as **7 seconds**, releasing pleasure hormones such as dopamine. When the vaping stops, nicotine levels in the body drop quickly, creating strong cravings to vape again. This cycle continues again and again and with continued vaping, can lead to changes in brain chemistry and increase nicotine tolerance. This cycle is known as **nicotine addiction**. As a result, the smoker experiences greater withdrawal symptoms between vapes or after they stop vaping. These withdrawal symptoms can be both physical and mental symptoms such as anxiety, fatigue, sweating, vomiting, and depression. Nicotine addiction can be prevented by not using tobacco products, such as e-cigarettes.

Are You At Risk of Nicotine Addiction?

Take a look at the five statements below and decide if you either **agree** or **disagree** based on your own experiences. If you answer "**agree**" to any of the statements below, it may mean that you are at risk of nicotine addiction. The good news is, there are people and resources available to help you on your journey to being nicotine-free.

1. I feel that I must use my preferred e-cigarette/tobacco product in the morning before school/work.
2. I feel a craving, like hunger, when I have not used an e-cigarette product in a while.
3. I use e-cigarettes/tobacco products even when I have a cold or another illness.
4. When I cannot use e-cigarettes/tobacco, I spend time thinking about and planning when I can use it next.
5. When I try to quit or take a break from e-cigarettes/tobacco for a while, I feel terrible.

1. American Lung Association
2. Adapted from InDepth, American Lung Association

Getting Started

What to Expect When You Stop Using Nicotine

1-2 Hours
Nicotine cravings begin

10 Hours
Possible changes in mood, such as feeling anxious, irritable, depressed, or having difficulty concentrating

24 Hours
Possible increase in appetite that can last for several weeks or longer

1 Week
Possible difficulty sleeping in the first week of nicotine withdrawal but adjusts after 1-2 weeks

2-4 Weeks
Less irritability and improved concentration

Know Your WHY and Plan Ahead

A good way to get started in quitting vaping is to know your "Why?". Why did you start vaping, why do you continue to vape, and why do you want to quit? Keeping these in mind can help you be mindful of your current vaping habits. Everyone's journey is different, but here are some useful tips to quit:

- Plan Ahead and Decide When You Will Quit
- Know What to Expect When You Quit
- Talk to Your Family/Friends/Support Group About Your Plan to Quit
- Avoid Your Triggers
- Use Resources Available Such As Quit Apps and QuitLines
- Keep Trying and Don't Be Discouraged if You Vape Again

Know your Triggers

Triggers are the things that make you want to vape, like dealing with stress or seeing someone else vape. Knowing your triggers and planning a way to cope other than vaping can help you stick with your plan to quit. Common vaping triggers include stress, boredom, feeling sad or angry, being around someone who is vaping, or being used to vaping at a certain time of day.

How to Cope

As you begin your journey to being vape-free, knowing and practicing healthy ways of coping when you feel like vaping again or experience a trigger is key. When you are feeling tempted, try the following coping technique: Use Something, Think Something, Do something, and Reward Yourself.

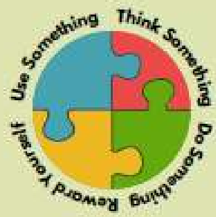

Use Something	Think Something	Do Something	Reward Yourself
Chew Gum	Meditate	Exercise	Set Small Goals and reward yourself/Celebrate when you reach them
Drink Water	Do a Crossword	Take a Nap	

Resources are Available to Help You Quit for Good

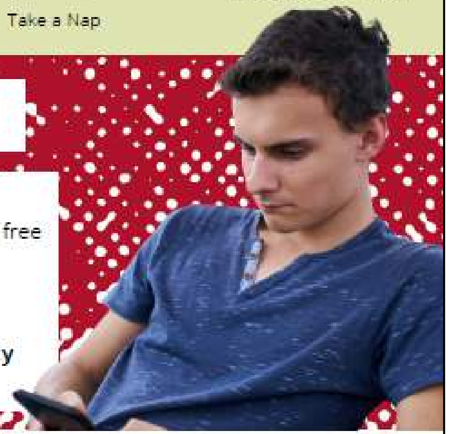

SmokeFree Teen ● www.teen.smokefree.gov
This website provides more information about how to stop vaping and offers free resources.

SmokeFreeTXT Program ● Text "QUIT" to 47848
Free texting program designed to help you quit vaping.

quitSTART ● available for free from the Apple Store and Google Play
Free app designed to give you the tools and support to quit vaping.

UNMC T.E.A.M.

This curriculum was developed by UNMC and TEAM. These vaping prevention materials are provided to participating schools at no cost. Please do not distribute this curriculum to other schools without our permission.

Appendix Table 1. Sample Characteristics of Current E-cigarette Users, United States, January 2022, (N= 361).

Age (years), Mean (SD)	361	34 (11.4)
	n	%
Sex		
Female	207	58.3
Male	148	41.7
Race/Ethnicity		
NH_Whites	258	72.3
NH_Blacks	20	5.6
Hispanics	38	10.6
Other	41	11.5
Education		
High School or Less	56	15.5
Some College	163	45.2
College Graduate	142	39.3
Income ($)		
<25,000	63	17.5
25,000-49,999	96	26.7
50,000-74,999	79	21.9
75,000-99,999	55	15.3
100,000+	67	18.6
Personal financial Situation		
Live comfortably	252	69.8
Just meet or Don't meet	109	30.2
Urban Status		
Rural	99	27.4
Urban	262	72.6
Sexual orientation status		
Heterosexual	271	75.1
Sexual minority	90	24.9
Other Tobacco Use		
Non Current	207	57.3
Current	154	42.7
Other Drug Use		
Non Current	73	20.2
Current	288	79.8
Current E-cigarette Use		
Some day	264	73.1

Daily	97	26.9
E-cigarette Devices Used		
Vape Pen	101	28.0
JUUL or Cartridge	95	26.3
Disposable	58	16.1
Others	107	29.6
Flavored Used		
Flavorless or Tobacco Flavored	59	16.3
Mint or Menthol	88	24.4
Sweet	132	36.6
Ice	48	13.3
Others	34	9.4
Past 6-month Quit Attempts		
No	252	70.0
Yes	108	30.0

Abbreviations, SD: Standard Deviation, NH: non-Hispanic.

Appendix Table 2. Evaluation of Vaping Cessation Infographics, United States, January 2022, (N = 361)

**Learn e-cigarettes**[a]	Overall	Sex: Female	Sex: Male	P-value[f]	Race/ethnicity: NH Whites	NH Blacks	Hispanics	Other	P-value[f]	E-cigarette Use: Some day	Daily	P-value[f]
Strongly Disagree	30 (8.5%)	14 (7%)	15 (10.2%	0.23	21 (8.3%)	4 (21.1%)	2 (5.3%)	3 (7.5%)	0.20	25 (10.1%)	5 (4.8%)	0.01
Disagree	118 (33.4%)	64 (32%)	53 (36.1%)		88 (34.9%)	3 (15.8%)	15 (39.5%)	10 (25%)		88 (35.6%)	30 (28.6%)	
Agree	165 (46.7%)	95 (47.5%)	68 (46.3)		115 (45.6%)	9 (47.4%)	15 (39.5%)	25 (62.5%)		114 (46.2%)	50 (47.6%)	
Strongly Agree	40 (11.3%)	27 (13.5%)	11 (7.5%)		28 (11.1%)	3 (15.8%)	6 (15.8%)	2 (5%)		20 (8.1%)	20 (19%)	
Mean score, SD[g]	2.6 (0.8)	2.7 (0.8)	2.5 (0.8)		2.6 (0.8)	2.6 (1)	2.7 (0.8)	2.7 (0.7)		2.5 (0.8)	2.8 (0.8)	

**Say no to e-cigarettes**[b]	Overall	Sex: Male	Sex: Female	P-value[f]	Race/ethnicity: NH_Whites	NH_Blacks	Hispanics	Other	P-value[f]	E-cigarette Use: Some day	Daily	P-value[f]
Strongly Disagree	56 (15.8%)	35 (17.3%)	18 (12.2%)	0.25	43 (16.9%)	6 (31.6%)	0	7 (17.5%)	N/A[g]	44 (17.7%)	12 (11.4%)	0.02
Disagree	168 (47.3%)	100 (49.5%)	66 (44.9%)		126 (49.6%)	7 (36.8%)	17 (44.7%)	16 (40%)		126 (50.6%)	42 (40%)	

Agree	107 (30.1%)	56 (27.7%)	51 (34.7%)		67 (26.4%)	6 (31.6%)	17 (44.7%)	15 (37.5%)		64 (25.7%)	42 (40%)	
Strongly Agree	24 (6.8%)	11 (5.4%)	12 (8.2%)		18 (7.1%)	0	4 (10.5%)	2 (5%)		15 (6%)	9 (8.6%)	
Mean score, SD[g]	2.3 (0.8)	2.2 (0.8)	2.4 (0.8)		2.2 (0.8)	2 (0.8)	2.7 (0.7)	2.3 (0.8)		2.2 (0.8)	2.5 (0.8)	

**Less likely to vape now**[c]		Sex			Race/ethnicity					E-cigarette Use		
	Overall	Male	Female	P-value[f]	NH Whites	NH Blacks	Hispanics	Other	P-value[f]	Some day	Daily	P-value[f]
Strongly Disagree	89 (25.1%)	50 (24.9%)	36 (24.5%)	0.37	69 (27.3%)	9 (47.4%)	3 (7.9%)	8 (20%)	N/A[g]	73 (29.4%)	16 (15.2%)	<.0001
Disagree	189 (53.4%)	112 (55.7%)	74 (50.3%)		136 (53.8%)	7 (36.8%)	23 (60.5%)	20 (50%)		138 (55.6%)	50 (47.6%)	
Agree	64 (18.1%)	31 (15.4%)	33 (22.4%)		41 (16.2%)	3 (15.8%)	8 (21.1%)	11 (27.5%)		30 (12.1%)	34 (32.4%)	
Strongly Agree	12 (3.4%)	8 (4%)	4 (2.7%)		7 (2.8%)	0	4 (10.5%)	1 (2.5%)		7 (2.8%)	5 (4.8%)	
Mean score, SD[g]	2.0 (0.8)	2.0 (0.8)	2.0 (0.8)		1.9 (0.7)	1.7 (0.7)	2.3 (0.8)	2.1 (0.8)		1.9 (0.7)	2.3 (0.8)	

**Better Understand Hidden Information**[d]	Overall	Sex			Race/ethnicity					E-cigarette Use		
		Male	Female	P-value[f]	NH Whites	NH Blacks	Hispanics	Other	P-value[f]	Some day	Daily	P-value[f]
Strongly Disagree	39 (11.2%)	22 (11.2%)	15 (10.4%)	0.71	34 (13.7%)	2 (10.5%)	0	3 (7.5%)	N/A[g]	29 (12%)	10 (9.6%)	0.47
Disagree	84 (24.2%)	52 (26.4%)	31 (21.5%)		57 (23%)	5 (26.3%)	9 (25%)	13 (32.5%)		63 (26%)	21 (20.2%)	
Agree	186 (53.6%)	102 (51.8%)	83 (57.6%)		130 (52.4%)	8 (42.1%)	21 (58.3%)	23 (57.5%)		126 (52.1%)	59 (56.7%)	
Strongly Agree	38 (11%)	21 (10.7%)	15 (10.4%)		27 (10.9%)	4 (21.1%)	6 (16.7%)	1 (2.5%)		24 (9.9%)	14 (13.5%)	
Mean score, SD[g]	2.6 (0.8)	2.6 (0.8)	2.7 (0.8)		2.6 (0.9)	2.7 (0.9)	2.9 (0.6)	2.6 (0.7)		2.6 (0.8)	2.7 (0.8)	

[a]: I learned things I did not already know about e-cigarettes.
[b]: I feel I would be more confident to say "No" if someone were to offer me an e-cigarette or invite me to vape.
[c]: I will be less likely to vape now.
[d]: I can better understand hidden messages in vaping ads, especially those targeted at young people
[e]: 4-level response options ranging from “strongly disagree” (coded 1) to “strongly agree” (coded 4).
[f]: Chi-square tests were conducted to compare subgroup evaluations of vaping cessation infographics.
[g] N/A = Not Applicable; SD = Standard Deviation.

© 2026 Dai H.D. et al.
